# Louse-borne relapsing fever (*Borrelia recurrentis* infection)

**DOI:** 10.1017/S0950268819000116

**Published:** 2019-03-01

**Authors:** David A. Warrell

**Affiliations:** Nuffield Department of Clinical Medicine, University of Oxford, Oxford, UK

## Abstract

Louse-borne relapsing fever (LBRF) is an epidemic disease with a fascinating history from Hippocrates’ times, through the 6th century ‘Yellow Plague’, to epidemics in Ireland, Scotland and England in the 19th century and two large Afro-Middle Eastern pandemics in the 20th century. An endemic focus persists in Ethiopia and adjacent territories in the Horn of Africa. Since 2015, awareness of LBRF in Europe, as a re-emerging disease, has been increased dramatically by the discovery of this infection in dozens of refugees arriving from Africa.

The causative spirochaete, *Borrelia recurrentis*, has a genome so similar to *B. duttonii* and *B. crocidurae* (causes of East and West African tick-borne relapsing fever), that they are now regarded as merely ecotypes of a single genomospecies. Transmission is confined to the human body louse *Pediculus humanus corporis*, and, perhaps, the head louse *P. humanus capitis*, although the latter has not been proved. Infection is by inoculation of louse coelomic fluid or faeces by scratching. Nosocomial infections are possible from contamination by infected blood. Between blood meals, body lice live in clothing until the host's body temperature rises or falls, when they seek a new abode.

The most distinctive feature of LBRF, the relapse phenomenon, is attributable to antigenic variation of borrelial outer-membrane lipoprotein. High fever, rigors, headache, pain and prostration start abruptly, 2–18 days after infection. Petechial rash, epistaxis, jaundice, hepatosplenomegaly and liver dysfunction are common. Severe features include hyperpyrexia, shock, myocarditis causing acute pulmonary oedema, acute respiratory distress syndrome, cerebral or gastrointestinal bleeding, ruptured spleen, hepatic failure, Jarisch–Herxheimer reactions (J-HR) and opportunistic typhoid or other complicating bacterial infections. Pregnant women are at high risk of aborting and perinatal mortality is high.

Rapid diagnosis is by microscopy of blood films, but polymerase chain reaction is used increasingly for species diagnosis. Severe falciparum malaria and leptospirosis are urgent differential diagnoses in residents and travellers from appropriate geographical regions.

High untreated case-fatality, exceeding 40% in some historic epidemics, can be reduced to less than 5% by antibiotic treatment, but elimination of spirochaetaemia is often accompanied by a severe J-HR.

Epidemics are controlled by sterilising clothing to eliminate lice, using pediculicides and by improving personal hygiene.

## Introduction

Louse-borne relapsing fever (LBRF) is a classic epidemic disease, associated with war, famine, refugees, poverty, crowding and poor personal hygiene. After a long history, recorded over many centuries, it is now largely confined to the Horn of Africa, while retaining its potential to cause future epidemics when conditions become conducive. It was a familiar infection in Europe and North America until the end of the 19th century after which it was forgotten. However, the recent surge of refugees from Africa arriving in European countries has brought this fascinating disease back into the view of the medical profession and has stimulated new research into its cause, *Borrelia recurrentis*, and its vector, the human body louse.

## Aetiology [1]

LBRF is caused by *B. recurrentis*, a large, loosely coiled, motile spirochaete (family Spirochaetaceae, that also includes *Treponema*), with tapering ends, 12–22 μm long and 0.2–0.6 μm thick, with an average wavelength of 1.8 μm, an amplitude of 0.8 μm and 8–10 periplasmic flagella [2]. They divide by transverse binary fission. *B. recurrentis* can be cultured on chick chorioallantoic membrane, and maintained in rodents [1]. Strains of immunodeficient mice (SCID lacking B and T cells, and SCID BEIGE lacking B, T, and NK cells) have been proposed as an animal model of LBRF [3]. *B. recurrentis* can be cultured *in vitro* using Barbour-Stoenner-Kelly (BSK-II) medium [4], BSK-H supplemented with heat-inactivated 10% rabbit serum and modified-Kelly-Pettenkofer (MKP) medium supplemented with 50% fetal calf serum [5]. BSK medium supports rapid initial borrelial growth but this is followed by cell deformation and death, whereas MKP medium appears to improve isolation rate, morphology and motility [6].

Unlike other bacteria, borreliae have a fragmented genome consisting of a linear chromosome, 1–15 linear plasmids and 1–9 circular plasmids. *B. recurrentis* has the simplest genome of all, composed of one linear chromosome and only seven linear plasmids, and only 990 protein coding genes. It shows low genetic variability [5]. Genomes of *B. recurrentis* and *B. duttonii* are identical except that in *B. recurrentis* 30 genes or gene families of *B. duttonii* are either absent or damaged. This has been cited as evidence that *B. recurrentis* has a decaying genome and is only a strain or subset of *B. duttonii* that adapted rapidly to louse-transmission with genome reduction [7]. *B. recurrentis* lacks RecA and RadA proteins that are responsible for DNA repair. The average nucleotide identity between the African borreliae, *B. crocidurae*, *B. duttonii* and *B. recurrentis*, is 99%, suggesting that they are merely ecotypes of the same genomospecies ‘*B. africana*’ [8].

## Transmission

Unlike most borreliae, transmission of *B. recurrentis* is restricted to one vector, the human body louse *Pediculus humanus corporis*, and, perhaps, the head louse *P. humanus capitis*. Although *B. recurrentis* has been identified in head lice, including those infesting pygmies in the Republic of Congo, outside the currently recognised geographical distribution of LBRF [9], transmission by them has not yet been confirmed. Body lice, unlike head lice, retreat from the skin after feeding to hide and lay their eggs in clothing seams rather than on hair shafts. In Addis Ababa, one old man was found to be harbouring more than 21 500 lice in his clothes [10]. Lice are obligate haematophagous human ectoparasites that ingest borreliae in their blood meal [11]. They are intolerant of deviations in human body temperatures caused by fever, climatic exposure or death, or when infested clothing is discarded. Then, they find a new host to whom borreliae can be transmitted. Coelomic fluid from a crushed louse, or louse faeces infected with *B. recurrentis*, is inoculated through broken skin, or intact mucous membranes such as the conjunctiva, by scratching. Blood transfusion, needlestick injuries and contamination of broken skin by infected blood are potential causes of nosocomial infections [12]. Since lice, unlike ticks, cannot infect their progeny, they do not act as reservoirs. Transplacental infection has been confirmed in a mouse model of *B. duttonii* infection [13] and there are reports of congenital infection by *B. hermsii* and other tick-borne spirochaetes [14]. There is no known animal reservoir, and so persistence of infection between epidemics can only be through mild or asymptomatic human infections.

## Epidemiology and historical background

Human disasters created by war, forced migrations, poverty, famine, breakdown of personal hygiene and seasonal spells of cold, wet weather, promote crowding and increase the risk of infestation by body lice and the transmission of LBRF, louse-borne typhus, trench fever and other louse-borne diseases. LBRF can be identified in historical descriptions of disease epidemics by the repeated recurrences of fever between asymptomatic periods of 4–7 days and by two typical symptoms, jaundice and bleeding. The earliest convincing description of this disease was given by Hippocrates in the 5th century BC in the North Aegean island of Thasos: ‘The great majority (of sufferers) had a crisis on the sixth day, with an intermission of six days followed by a crisis on the fifth day after the relapse.’ Other features typical of LBRF were severe rigors, jaundice, profuse epistaxes and tendency to precipitate abortion [15, 16]. MacArthur has argued convincingly that the ‘Yellow Plague’ that engulfed Europe in 550 AD, in the wake of the Justinian plague, and the famine fevers of the 17th and 18th centuries in Ireland and elsewhere, whose defining feature was jaundice, were predominantly LBRF [16].

Recently, a historical genome of *B. recurrentis* was recovered from the skeleton of a young woman found during the excavation of a graveyard near St. Nicolay's Church in Oslo. Radiocarbon dating suggested that its age was AD 1430–1465. The mediaeval European genome displayed an ancestral oppA-1 gene, and gene loss in antigenic variation sites (variable short and long membrane protein genes) that translated into a genome reduction of 1.2% of the pan-genome, and 5.1–21% of the affected plasmids, perhaps associated with increased virulence but a reduced number of relapses [17].

In Dublin in 1770, Rutty described ‘a fever altogether without the malignity attending (typhus), of six or seven days duration, terminating in a critical sweat…in this the patients were subject to a relapse, even to a third or fourth time, and yet recovered’ [18]. In Edinburgh in 1843, Craigie distinguished LBRF from typhus and coined the name ‘relapsing fever’ [19]. Henderson detailed the differences between the two infections [20]. In Britain, in the 19th century, LBRF featured prominently in Charles Murchison's treatise on continued fevers. He commented on its intermittent appearance and truly epidemic nature: ‘So completely did relapsing fever disappear from Britain after 1828 that when, after an interval of fourteen years, it again showed itself as an epidemic in 1843, the junior members of the profession failed to recognize it and it was regarded as a new disease’ [21]. Obermeier saw spirochaetes, now recognised as *B. recurrentis*, in the blood of febrile patients in Berlin in 1866 [22]. Transmission by human body lice was proved by Mackie in 1907 [23].

In the 20th century, from 1903 to 1936, a huge pandemic swept across North Africa, the Middle East and Africa, causing an estimated 50 million cases with 10% mortality. A second epidemic in 1943–46 created 10 million cases [11].

An endemic focus persists in the Horn of Africa [24]. In cold, wet weather, impoverished people with louse-infested clothes crowd together for warmth and shelter. These indigent, malnourished street-dwellers, day workers (casual labourers), usually young men and prisoners, are the most vulnerable to infection. In the Ethiopian highlands there are annual epidemics of thousands of cases coinciding with the rains. Outbreaks have also occurred in Somalia. In Rumbek County, South Sudan, in 1999–2000, there were 20 000 cases with some 2000 deaths, 580 in January 1999 alone [25]. In 1985, in Chavin District if Ancash Province in the Peruvian Andes at altitudes above 3800 m, 60 clinical cases were reported among louse-infested villagers, 36 with *B. recurrentis* in their blood films [26]. More recently in Calca Province in the Urubamba Valley of Peru, antibodies to *B. recurrentis* have been found in two of 194 villagers [27]. The discovery of *B. recurrentis* in head lice in Congolese pygmies raises the possibility of other undiscovered human reservoirs [9].

### *B. recurrentis* infection in African refugees arriving in Europe

Since July 2015, LBRF has been diagnosed in almost 100 mainly young male refugees, who arrived in several European countries, most in Italy and Germany, seeking asylum after travelling from Ethiopia, Eritrea, Somalia and other African countries, usually through Libya [28–34]. It is the most frequently reported infection in Eritrean immigrants [34]. It seems likely that many other cases may have gone undetected and unreported [30]. Most of the patients were from the Horn of Africa, but the duration of their symptoms suggested that the majority had been infected in Libya. However, two cases diagnosed in Turin, Italy were long-term residents who shared accommodation with recently arrived immigrants, suggesting the possibility of autochthonous infection [31]. Some, from African countries not endemic for LBRF such as Mali, were probably infected during their journey, in crowded transit hostels in Libya, Italy or elsewhere [35]. LBRF was largely unknown in Germany and other European countries before its re-emergence after 2015 [29], but there is now the possibility that it might become re-established in some impoverished and crowded immigrant populations in parts of Europe [31].

## Pathophysiology and pathology

### The relapse phenomenon

Attacks of relapsing fever end abruptly when specific bactericidal immunoglobulin M antibodies generated by the B1b cell subset lyse spirochaetes in the blood, independently of complement and T cells. Between relapses, spirochaetes may persist extracellularly in spleen, liver, kidneys, eye and other sites. Relapses, accompanied by spirochaetaemia, are explained by antigenic variation, which has been studied in depth in the North American tick-borne pathogen, *B. hermsii* [36]. Silent gene sequences from an archive stored in plasmids are transposed to one end of an expression linear plasmid where their recombination leads to synthesis of a new variable major outer membrane lipoprotein (vmp) [36]. The new external membrane allows borreliae to evade the host's humoral immune response until antibodies are generated against the new serotypic vmp antigen, explaining the sequential emergence of borreliae expressing different vmps during the course of an untreated infection. Stimulation of the massive release of tumour necrosis factorα (TNF-α) at the start of the Jarisch–Herxheimer reaction (J-HR) to antibiotic treatment in LBRF is also due to vmp [37]. *B. recurrentis* is protected from the host's innate immunity by expressing receptors that selectively bind C4bp and C1-Inh, the endogenous regulators of the classical and lectin complement pathway, HcpA protein that, by binding plasmin, decreases C3b deposition and a specific receptor for the serum-derived complement inhibitor of the alternative pathway, CFH [38]. These mechanisms allow evasion of lysis by complement activation. Another possible protective mechanism is rosetting of erythrocytes around some Borrelia spirochaetes that masks or excludes them by steric hindrance, from host antibody. However, although *B. crocidurae* and *B. duttonii* induce rosetting, *B. recurrentis* do not [39].

### Pathophysiology

The spontaneous crisis that terminates untreated attacks, and the J-HR induced by antibiotic treatment, show pathophysiological features of a classic tri-phasic endotoxin reaction. *B. recurrentis* outer membrane vmps stimulate monocytes to produce TNF-α through NF-κB [37]. There are transient marked elevations in plasma concentrations of TNF-α, interleukin (IL)-6, IL-8 and IL-1β [40, 41]. The massive burst of cytokines is triggered by phagocytosis of spirochaetes opsonised by the antibiotic. Penicillin binding results in large surface blebs on the spirochaetes which are then phagocytosed by neutrophils in the blood and by the spleen. *In vitro*, surface contact with spirochaetes induces mononuclear leucocytes to produce inflammatory cytokines and thromboplastin, causing fever and disseminated intravascular coagulation [42]. Kinins may be released during the J-HR. The profound leucopenia that develops during the reaction reflects sequestration rather than leucocyte destruction. Spirochaetes may be found in liver, spleen, myocardium and brain. Thrombocytopenia rather than vasculitis causes the petechial rash. Cardiorespiratory and metabolic disturbances result from persistent high fever, accentuated by the J-HR or spontaneous crisis [43].

### Pathology [44, 45]

**Fig. 1.**
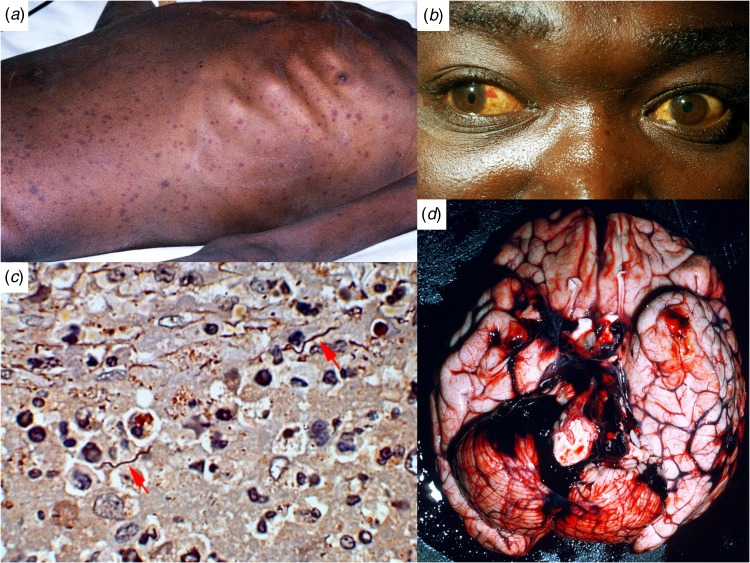
Ethiopian patients with LBRF. (A) Profuse petechial rash on the trunk in an emaciated patient with complicating infection with *Salmonella enterica* serovar Typhi (*S*. Typhi). (B) Subconjunctival haemorrhages and jaundice indicative of hepatocellular damage, thrombocytopenia and coagulopathy. (C) *B. recurrentis* spirochaetes arrowed (silver stain) in the splenic pulp. (D) Cerebral haemorrhage on the 6th day of illness, a common cause of death in patients with LBRF.

Spirochaetes are mainly confined to the lumen of blood vessels, but tangled masses occur in splenic miliary abscesses and (Fig. 1C) infarcts as well as within the central nervous system adjacent to haemorrhages. A perivascular histiocytic interstitial myocarditis, found in the majority of fatal cases, may be responsible for conduction defects, arrhythmias and myocardial failure resulting in sudden death. Splenic rupture with massive haemorrhage, cerebral haemorrhage and hepatic failure are other causes of death [46]. There is hepatitis with patchy midzonal haemorrhages and necrosis, meningitis and perisplenitis. Serosal cavities and surfaces of viscera are studded with petechial haemorrhages and sometimes massive pulmonary haemorrhages, reminiscent of leptospirosis. Thrombi are occasionally found occluding small vessels, but the peripheral gangrene, that is a feature of louse-borne typhus (*Rickettsia prowazekii* infection) [47, has not been reported in LBRF [12].

## Symptoms and signs [12]

The incubation period is 4–18 (average 7) days. The attack starts abruptly with a fever that increases to nearly 40 °C in a few days, accompanied by rigors. Early symptoms include headache, dizziness, nightmares, generalised aches and pains; affecting especially the lower back, knees and elbows; anorexia, nausea, vomiting and diarrhoea. Upper abdominal pain, cough and epistaxis develop later. Prostration and confusion are the rule. The commonest sign is hepatic tenderness (in about 60%) and enlargement (50%). Splenic tenderness and enlargement are less common. Jaundice is found in seven to >70% of patients [12]. A petechial or ecchymotic rash, particularly involving the trunk (Fig. 1A), is seen in 2% to 80% of patients [12]. It must be distinguished from the maculo-papular or petechial rash of louse-borne typhus. Subconjunctival haemorrhages (Fig. 1B) and epistaxis (25%) are common, haemoptysis, gastrointestinal bleeding and retinal haemorrhages less so. Many patients have myalgia. Meningism occurs in about 40% of patients. Neurological symptoms are less common than in tick-borne relapsing fevers: cranial nerve lesions, monoplegias, flaccid paraplegia and focal convulsions. Untreated attacks resolve by crisis after 4–10 (average 5) days, followed by an afebrile remission of 5–9 days, succeeded by up to five relapses of diminishing severity, during which there may be epistaxis but no petechial rashes.

Pregnant women are especially susceptible to severe disease and premature labour, and still births are frequent. In tick-borne relapsing fever caused by *B. duttonii*, intrauterine growth retardation, placental damage and inflammation, impaired fetal circulation and maternal anaemia have been described. Spirochaetes frequently cross the placenta, resulting in congenital infections [13].

## Clinical in refugees arriving in Europe

Clinical findings and results of laboratory investigations in 55 refugees [48] have been compared with those in 62 Ethiopian patients studied in Addis Ababa in the late 1960s [12]. Symptoms such as fever, headache, myalgias, abdominal pain and vomiting were common in both groups, but, among the refugees, jaundice was more often reported (51% *vs.* 34%). Bleeding (8.9% *vs.* 23%), meningism (5.5% *vs.* 39%), J-HRs (62% *vs.* 100%) and fatalities (1.8% *vs.* 4.8%) were less common. Levels of C-reactive protein [median 284 (55–440.9) mg/dl] and procalcitonin [13.93 (0.95–62.1) ng/ml] were raised in the refugees [48]. Twenty percent of the refugees had raised serum creatinine concentrations [2.4 (0.9–4.7) mg/dl], indicating renal impairment [48]. Body lice were recovered from 22% of the refugees and in others there were scratch marks suggesting infestation [48].

Children: In Sheshamane, Ethiopia, children younger than 15-years-old were compared with adults [49]. Clinical features in children resembled those in adults but were generally less severe and less frequent. Headache (40%), dizziness (39%), abdominal cramps (17.4%), vomiting (23.8%), cough (27.6%), musculoskeletal pain (30.5%), petechial rash (1.9%) and bleeding (3.8%) were all less common in the children [49]. In a later study from the same hospital, fever, headache, dizziness and musculoskeletal pains were said to be the commonest symptoms [50]. A study of infants and children in Arsi Region, Ethiopia, found that the common clinical features were fever (100%), headache (84.5%), chills (74%), abdominal pain (51%), epistaxis (20%), hepatomegaly (26%), splenomegaly (14%), petechial rash (34%) and jaundice (10%). Pneumonia (14%) and central nervous system involvement (10%) were common complications. J-HRs occurred in 61%. Case fatality was 1.9% [51].

### Prognosis

Case fatalities between 30% and 70% have been reported in untreated patients during major historic epidemics, but in treated cases, on average, 2–6% will die [12]. In an outbreak in Arsi Zone, Ethiopia in 2016, the case-fatality was 13% [52]. Reported case fatalities in children range from 1.9% to 5.5%. In one series of 154 children (<15 years) in Ethiopia, overall case fatality rate was 2.4%, less than in adults (13.2%) [50].

### Severe louse-borne relapsing fever

Clinical features associated with a bad prognosis include coma; shock; hyperpyrexia; myocarditis with acute pulmonary oedema [53]; acute respiratory distress syndrome; hepatic failure; ruptured spleen and haemostatic failure from thrombocytopenia liver damage and disseminated intravascular coagulation leading to intracranial (Fig. 1D), massive gastrointestinal, pulmonary or peripartum haemorrhage [12, 43, 50]. Complicating co-infections such as dysentery, salmonellosis, typhoid, typhus, tuberculosis, bacterial pneumonia, visceral leishmaniasis and malaria increase mortality [12, 45].

### Spontaneous crisis and J-HR

An impending crisis on about the fifth day of the untreated illness, or a J-HR about 1 to 2 h after antibiotic treatment, is signalled by restlessness and apprehension, followed by distressingly intense rigors lasting 10 to 30 min [12, 43]. During this chill phase, temperature, respiratory and pulse rates, and blood pressure rise steeply, with associated delirium, gastrointestinal symptoms, cough and limb pains. Fatal hyperpyrexia may occur. The flush phase, characterised by profuse sweating, a fall in blood pressure, and a slow decline in temperature, may last for many hours. During this period, patients may collapse and die if they stand up or may develop progressive and intractable hypotension, especially if they suffer acute myocardial failure attributable to borrelial myocarditis [53]. Treatment with intravenous tetracycline carries the highest risk of provoking a J-HR, reaching 100% in some studies [12]. Low-dose or slow-release penicillin causes fewer reactions but may not prevent relapses (see below). In children, J-HRs are less common.

## Laboratory investigations [12]

Circulating spirochaete densities may exceed 500 000/mm^3^ of blood. Patients commonly have a moderate normochromic anaemia with neutrophil leucocytosis. The spontaneous crisis and J-HR are marked by leucopenia. Thrombocytopenia is usual and there is a mild coagulopathy (raised prothrombin time and INR) with evidence of increased fibrinolysis (increased fibrinogen degradation products or D-dimer). Raised serum concentrations of aminotransferases, alkaline phosphatase, direct and total bilirubin and low albumin suggest hepatocellular damage. Mild renal impairment is common. The cerebrospinal fluid shows a lymphocyte or neutrophil pleocytosis without detectable spirochaetes.

Electrocardiographical (ECG) evidence of myocarditis includes prolongation of the QTc interval, T-wave abnormalities and ST-segment depression with transient acute right heart strain after the J-HR and various arrhythmias [53]. Chest radiographs are usually clear but may show pulmonary oedema or pneumonic consolidation.

## Diagnosis

### Microscopy

The possibility of rapid bed-side diagnosis makes LBRF a satisfying disease for the clinician. Thick and thin blood films should be taken while patients are febrile and stained with Giemsa, May-Grünwald Giemsa, Wright, Wright-Giemsa, Field's, or Diff-Quick stains, or examined under dark-field. Positivity thresholds of thin and thick smear blood are respectively estimated at 10^5^ and 10^4^ spirochaetes per millilitre of blood [54]. A two-stage centrifugation concentration method has been described [55]. Quantitative buffy coat technique (acridine orange) is also possible. The higher and more persistent spirochaetaemia in LBRF makes microscopic diagnosis more reliable than in the other borrelioses. Exflagellating *Plasmodium vivax* microgametes may be mistaken for spirochaetes (‘pseudoborreliosis’) [56], but microfilariae are far too large to cause confusion.

### Polymerase chain reaction (PCR)

An important break-through has been the development of a multiplex real-time PCR (MR-TPCR) method, to differentiate the four main *Borrelia* species in Africa [57]. It targets the 16S rRNA gene (detecting all four species); glpQ gene (*B. croidurae*); recN gene (*B. duttonii*/*B. recurrentis*) and recC gene (*B. hispanica*). The assay has a 100% sensitivity and specificity for *B. duttonii*/*B. recurrentis*, but could not discriminate between these two species because of their very close genetic and genomic proximity that suggests they may be a single species [7, 8]. PCR detected 100 copies, proving to be more sensitive than the 10^3^–10^5^ borreliae/mL visible by microscopy. Among infected immigrants to Europe, PCR has proved a valuable method for confirming the species diagnosis of *B. recurrentis* [29–32], and has detected some microscopy-negative cases [29, 48]. PCR has been successfully introduced at a point-of-care laboratory in rural Senegal for diagnosis of *B. crocidurae* infections [58].

### Serology

Sera from patients with LBRF may give positive reactions with Proteus OXK, OX19 and OX2, which might suggest the diagnosis. False-positive serological responses for syphilis are found in in 5–10% of cases. Serology has generally proved unreliable and non-specific, but it has been improved by the use of the *glp*Q gene as a recombinant antigen [59], or monoclonal antibodies (to *B. crocidurae*) [60]. However, serology lacks sufficient specificity, is not commercially available, and may fail to detect acute infections [59, 60].

## Differential diagnosis

In a febrile patient in or from Africa, who has all the classic features of LBRF – jaundice, petechial rash, epistaxis, hepatosplenomegaly, thrombocytopenia, coagulopathy and elevated serum aminotransferases – severe falciparum malaria is the most urgent differential diagnosis. In the Horn of Africa, yellow fever and other viral haemorrhagic fevers such as Rift Valley Fever and viral hepatitis, rickettsial infections, especially louse-borne typhus which occurs in mixed epidemics with LBRF, must be considered. If there is evidence of acute kidney injury, leptospirosis is more likely. Co-infection of LBRF with leptospirosis was diagnosed by PCR in a refugee from East Africa who arrived in Italy [61]. Trench fever (*Bartonella quintana*), transmitted by lice, can also cause episodic recurrent fever with headache and pains in the shins, but it lacks the bleeding and jaundice of LBRF. In endemic areas, complicating bacterial infections, particularly typhoid, or coinfection with malaria should not be forgotten [12, 45]. In refugees diagnosed in Europe, *P. falciparum* malaria, sepsis, leptospirosis and meningitis have been cited as leading differential diagnoses [48].

## Treatment

### Antibiotics

Complete cure with prevention of relapses is achievable with a single oral dose of 500 mg tetracycline or 500 mg erythromycin stearate. However, vomiting is so common that parenteral treatment is more dependable. A single intravenous dose of 250 mg tetracycline hydrochloride or, for pregnant women and children, a single intravenous dose of 300 mg erythromycin lactobionate (children 10 mg/kg body weight) is effective. In mixed epidemics of LBRF and louse-borne typhus, a single oral dose of 100 mg doxycycline is effective [62]. Benzyl penicillin (300 000 units = 80 mg), procaine penicillin with benzyl penicillin (600 000 units) and procaine penicillin with aluminium monostearate (600 000 units), all by intramuscular injection, are often effective but may fail to prevent relapses [63]. Long-acting preparations clear spirochaetaemia slowly and the J-HR is protracted. Some experienced clinicians prefer to use a low initial dose of penicillin (adult dose, 100 000–400 000 units by intramuscular injection) in severe cases and pregnant women because they believe that the incidence and severity of the J-HRs will be less. In a randomised clinical trial of three regimens of intramuscular procaine penicillin and one of oral tetracycline in Gondar, Ethiopia, the incidence of J-HRs increased with increasing doses of penicillin, from 5.1% with 100 000 units to 31.1% with 400 000 units, and was 46.6% in patients treated with tetracycline [64]. Since fatalities (3.3%) were associated with J-HRs, the authors recommended that treatment be initiated with low dose penicillin, despite relapse rates that decreased from 45% to 9.4% from the lowest to highest doses of penicillin. There were no relapses after tetracycline treatment as had been described earlier [12]. The combination of penicillin on the first day of treatment, followed by tetracycline on the next day, deserves further study [65]. Chloramphenicol is effective in a single dose of 500 mg by mouth or intravenous injection in adults. It may not be available in some Western countries.

### Preventing the J-HR

Antibiotics, such as tetracycline, that rapidly eliminate spirochaetes from the blood and prevent relapses, often induce a severe, and rarely fatal, J-HR. Antibiotic treatment cannot be withheld in view of the high untreated mortality, especially as severe spontaneous crises, that occur in a large proportion of LBRF cases on or after the fifth day of fever, may also prove fatal. There is no conclusive evidence that the apparently milder reaction following slow-release penicillin, compared to tetracycline is any less dangerous [63, 64].

Pre-treatment with oral prednisolone may prevent the J-HR of early syphilis, but neither an oral dose of 3 mg/kg prednisolone given 18 h beforehand, nor an infusion of 3.75 mg/kg betamethasone, prevented the J-HR in LBRF [66]. Hydrocortisone in doses up to 20 mg/kg [66], paracetamol [42] and pentoxifylline [67] failed to prevent the J-HR. However, meptazinol, an opioid antagonist/agonist, diminished the reaction when given in a dose of 100 mg by intravenous injection [68, 69]. A polyclonal ovine Fab anti-TNF-α antibody infused for 30 min before treatment with intramuscular penicillin, effectively prevented or diminished the J-HR [70], but, unfortunately, has not been made available for use in the endemic areas. Recombinant human IL-10 was not effective [71].

### Supportive treatment

During spontaneous crisis or J-HR, hyperpyrexia must be actively prevented with antipyretics, vigorous fanning and tepid sponging. The prolonged ensuing flush phase poses dangers of hypotensive shock and postural hypotension and so patients must be nursed lying in bed for at least 24 h after treatment. Most patients are dehydrated and relatively hypovolaemic and adults may need 4 litres or more of isotonic saline intravenously during the first 24 h. Infusion should not be excessive, but carefully monitoring, by observing jugular venous or central venous pressures. Borrelial myocarditis predisposes to acute myocardial failure during the flush phase when there is a demand for a high cardiac output to sustain blood pressure in the face of systemic vasodilatation. Warning signs are symptoms of acute myocardial failure, a rise in central venous pressure above 15 cm H_2_O, and prolonged ECG QTc interval [53]. One mg of digoxin given intravenously over 5–10 min has proved effective in this situation [72]. Diuretics may worsen the circulatory failure by causing relative hypovolaemia in the presence of the intense vasodilatation. Oxygen should be given during the reaction, particularly in severe cases. Patients with prolonged prothrombin times should be treated with vitamin K. Heparin is not effective in controlling coagulopathy and should not be used. Complicating opportunistic infections (typhoid, salmonellosis, bacillary dysentery, tuberculosis, typhus, visceral leishmaniasis, malaria) must be anticipated and treated appropriately.

## Prevention and control of epidemics

For *B. burgdorferi*, an effective vaccine has been developed for dogs, but not yet for humans. However, there has been no interest in developing vaccines against relapsing fever borreliae.

Breaking louse transmission is essential for the control of an epidemic. Infested clothing should be deloused using heat (>60 °C) or washing at 52 °C for 30 min. Patients should be bathed with soap. Lice have developed some degree of resistance to the most commonly used topical pediculicides, including 10% dichloro-diphenyl-trichloroethane, lindane, 1% malathion, 2% temephos, 1% propoxur and 0.5% permethrin [11]. Head lice should be removed by washed or shaving although their role in LBRF is unproven. Separating infested clothes from wearers for 10 days starves lice to death at any ambient temperature [73].
